# Unraveling the Association Between Fibromyalgia and Irritable Bowel Syndrome: A Systematic Review

**DOI:** 10.7759/cureus.96801

**Published:** 2025-11-13

**Authors:** Roshitha S Bheemaneni, Priyanka Sakarkar, Aahana Nigam, Evangeline C Nwachukwu, Sandeep Sekar Lakshmisai, Lubna Mohammed

**Affiliations:** 1 Medicine, Gandhi Medical College, Secunderabad, IND; 2 General Surgery, Princess Royal University Hospital, Orpington, GBR; 3 Cardiology, Trinity College Dublin, Dublin, IRL; 4 Health Emergency Preparedness and Response, Nigeria Centre for Disease Control and Prevention, Abuja, NGA; 5 Medicine, SRM Prime Hospital, Chennai, IND; 6 Internal Medicine, Dr. VRK Women's Medical College, Aziznagar, IND

**Keywords:** fibromyalgia, fm, gut-brain axis, gut microbiome, ibs, irritable bowel syndrome, probiotics, serotonin

## Abstract

Fibromyalgia (FM) and irritable bowel syndrome (IBS) often occur together. Patients with FM and IBS present similar symptoms, such as pain and fatigue; this leads to a delay in diagnosis and management. This systematic review explored the shared pathophysiology of these conditions in adults, focusing on the roles of immune dysfunction, gut dysbiosis, neurotransmitter imbalances, and disturbances in the gut-brain axis. We searched five databases, PubMed, PubMed Central, Google Scholar, Cochrane, and ScienceDirect, for relevant free full-text English articles from 2015 to 2025. Ten studies were selected after screening, identification, and quality assessment, adhering to the Preferred Reporting Items for Systematic Reviews and Meta-Analyses (PRISMA) 2020 guidelines. Our review found that immune system dysregulation involves mast cells and pro-inflammatory cytokines that damage the gut barrier. The gut microbiome and neurotransmitter levels seem to have a reciprocal influence on each other, and their alteration contributes to pathogenesis, with an increase of certain species showing an association with symptom severity. Serotonin and tryptophan metabolism appear to have a crucial role in pain perception, particularly visceral hypersensitivity. Therapeutic strategies targeting the gut microbiome, such as probiotics and fecal microbiota transplantation, have potential but require further research. Overall, this review identified overlapping mechanisms of FM-IBS comorbidity, which can pave the way to effective and combined treatment approaches. Future research should explore gender distinctions in the mechanisms, medications that act on neurotransmitter receptors (especially serotonergic pathways), and the utility of fecal microbiota transplantation and probiotics.

## Introduction and background

Fibromyalgia (FM) is a chronic condition that causes widespread muscular pain, fatigue, and poor quality of sleep. Irritable bowel syndrome (IBS) is a chronic gastrointestinal illness that presents with abdominal pain and altered bowel habits. Their coexistence in patients leads to a higher frequency of FM flares, aggravation of gastrointestinal symptoms, and worsened quality of life [1,2]. Since both conditions are diagnosed by excluding other disorders through clinical evaluation and selective testing, their co-occurrence complicates the diagnosis. Diagnostic delays averaging two to five years for FM and one to three years for IBS are common [3-6]. Studies have shown that 28-59% of patients with FM develop IBS, while 32-77% of patients with an IBS diagnosis develop FM [7]. These variations may stem from methodological differences across studies, including variations in diagnostic criteria application, study population characteristics (particularly gender distribution, with both conditions showing two to three times higher prevalence in women), geographic location, and sample sizes [2].

FM is a complex pain disorder characterized by abnormal pain perception, musculoskeletal pain, fatigue, and cognitive and sleep disturbances [8]. The 2016 revisions to the American College of Rheumatology (ACR) fibromyalgia diagnostic criteria introduced the Widespread Pain Index (WPI) and Symptom Severity Scale (SSS) as key components for diagnosis [9]. The pathophysiology of FM is intricate, involving genetic predisposition, environmental stressors, neurotransmitter (NT) dysregulation, and alterations in neuroendocrine pathways. A metabolic dysfunction of tryptophan (Trp, a precursor to serotonin) has also been detected [10]. Recent studies are shedding light on the role of an altered gut microbiome in shaping this disease, particularly immune activation and disturbed metabolism of bacterial breakdown products [11].

IBS is a functional gastrointestinal disorder that accounts for at least 20% of outpatient gastroenterology referrals [8]. The diagnosis is established based on the Rome IV criteria, which describe IBS as abdominal pain that recurs, on average, at least one day in a week in the previous three months, along with two or more of the following: pain related to defecation, altered stool frequency, or change in stool appearance. The pathophysiology involves alterations in the gut microbiota and gut motility. In recent studies, the gut-brain axis (gut-brain axis is a bidirectional communication network linking the gastrointestinal tract and the central nervous system) has been postulated to play a major role, as visceral hypersensitivity and gut mucosal inflammation have been noted [12]. NTs serve as messengers between the enteric microbiome and the brain. Specifically, serotonin (5-hydroxytryptamine or 5-HT) is produced by enterochromaffin cells (ECs) in the gut and serotonergic neurons in the central nervous system (CNS). It is known to modulate peristalsis, along with secretory, vagal, vasodilatory, and nociceptive functions. An altered signaling of 5-HT is implicated in the pathophysiology of IBS [13].

Both diseases have common associated factors, such as stress, fatigue, hypersensitivity, pain, depression, and anxiety. The shared symptoms of these diseases and their co-occurrence suggest similarities in their pathophysiologic development. In particular, their shared mechanisms seem to involve NT dysregulation, inflammatory cells, neuroendocrine pathways, dysbiosis of gut microbiota, and the gut-brain axis [7,8]. The two key mechanisms proposed to link IBS and FM are central sensitization and disturbance of the gut-brain axis [2]. Central sensitization refers to a state where the central nervous system amplifies pain signals from normal stimuli, contributing to hypersensitivity in both conditions [2].

This systematic review aims to further study the correlation between the pathophysiological pathways of FM and IBS. A comprehensive understanding of the strong association between these diseases will urge clinicians to consider their combined presentation in patients, paving the way to timely diagnosis and management. The roles of inflammatory and immunological mechanisms, gut dysbiosis, and NT alterations will be explored, alongside potential therapies.

## Review

Methods

This systematic review was conducted following the Preferred Reporting Items for Systematic Reviews and Meta-Analyses (PRISMA) 2020 guidelines [14].

Eligibility criteria 

Inclusion criteria: English language studies published between 2015 and 2025 and with unrestricted access to their full text were selected. The 2015 cutoff reflects post-Rome IV (2016) and ACR revisions (2016), capturing microbiome-era advances. This review included research and review articles involving male and female participants aged 18 and upward. 

Exclusion criteria: Papers published in languages other than English were excluded. Papers lacking full text availability were also excluded. This was due to the lack of access to critically appraise these papers in their entirety, which would risk the reliability of this review. Papers published before 2015 were excluded to maintain relevance to the most recent advancements. Studies involving children and those reporting on animal experiments were excluded to focus on the human adults aged 18 and above. Study protocols and Mendelian randomization studies were excluded due to their lack of analysis, which was needed in this review.

Search Strategy and Databases

We utilized PubMed Central, PubMed, Google Scholar, ScienceDirect, and Cochrane. To ensure a comprehensive search, we employed a combination of Medical Subject Headings (MeSH) and keywords using the Boolean operators. These keywords included “irritable bowel syndrome”, “IBS”, “mucous colitis”, “fibromyalgia”, “fibromyositis”, “gut-brain axis”, “etiology”, “pathogenesis”, and “pathophysiology”. Detailed information regarding the search strategy can be found in Table 1.

**Table 1:** Number of articles identified from each database. MeSH =  Medical Subject Headings

	Search Strategy	Database used	Filters	Number of research papers
A	((((((Fibromyalgia[Title]) OR (Fibromyositis[Title])) AND (Gut brain axis)) )	PubMed	Last 10 years, free full text, English language, adults.	4
B	((Irritable bowel syndrome[Title]) OR (Mucous colitis[Title])) AND (Gut brain axis)	PubMed	Last 10 years, free full text, English language, adults.	34
C	(((Fibromyalgia[Title]) OR (Fibromyositis[Title])) AND (Irritable bowel syndrome[Title])) OR (mucous colitis[Title])	PubMed	Last 10 years, open access with PubMed links	16
D	(( "Fibromyalgia/etiology"[Mesh] OR "Fibromyalgia/pathology"[Mesh] OR "Fibromyalgia/physiopathology"[Mesh] )) AND ( "Irritable Bowel Syndrome/etiology"[Mesh] OR "Irritable Bowel Syndrome/pathology"[Mesh] OR "Irritable Bowel Syndrome/physiopathology"[Mesh] )	PubMed	Last 10 years, open access with PubMed links	11
E	(Fibromyalgia[Title] OR Fibromyositis[Title]) AND ("brain-gut axis"[MeSH Terms] OR ("brain-gut"[All Fields] AND "axis"[All Fields]) OR "brain-gut axis"[All Fields] OR ("gut"[All Fields] AND "brain"[All Fields] AND "axis"[All Fields]) OR "gut brain axis"[All Fields]) AND ("open access"[filter] AND "2015/03/03"[PubDate] : "2025/02/27"[PubDate])	PubMed Central	Last 10 years, open access with PubMed links	91
F	(Irritable bowel syndrome[Title] OR Mucous colitis[Title]) AND ("brain-gut axis"[MeSH Terms] OR ("brain-gut"[All Fields] AND "axis"[All Fields]) OR "brain-gut axis"[All Fields] OR ("gut"[All Fields] AND "brain"[All Fields] AND "axis"[All Fields]) OR "gut brain axis"[All Fields]) AND ("open access"[filter] AND "2015/03/03"[PDat] : "2025/02/27"[PDat])	PubMed Central	Last 10 years, open access with PubMed links	80
G	((IBS[Title]) OR (Irritable bowel syndrome[Title])) AND (Fibromyalgia[Title]) ( "Fibromyalgia/etiology"[Mesh] OR "Fibromyalgia/pathology"[Mesh] OR "Fibromyalgia/physiopathology"[Mesh] )) AND ( "Irritable Bowel Syndrome/etiology"[Mesh])	PubMed Central	Last 10 years, open access with PubMed links	16
H	"Irritable Bowel Syndrome/pathology"[Mesh] OR "Irritable Bowel Syndrome/physiopathology"[Mesh] ) pathology"[Mesh] OR "Irritable Bowel Syndrome/physiopathology"[Mesh] )	PubMed Central	Last 10 years, open access with PubMed links	10
I	“Fibromyalgia” “irritable bowel syndrome” “adults”	Cochrane (advanced search)	2015-2025	22
J	allintitle: Irritable Bowel Syndrome OR IBS AND Fibromyalgia	Google Scholar	2015-2025, first 100 records screened.	34
K	TITLE("Fibromyalgia") AND TITLE("IBS" OR "Irritable Bowel Syndrome") "adults" AND "pathogenesis"OR"Pathophysiology"	Science Direct	2015-2025, medicine, open access and open archive	24

Study Selection 

Two reviewers screened and assessed the articles based on eligibility criteria and relevance to the review. Disagreements were resolved through thorough discussion, and another reviewer was brought in when needed. We used Microsoft Excel (Microsoft Corporation, Redmond, Washington, United States) to track decisions.

Data Extraction and Synthesis 

Data extraction was carried out by four authors in two teams independently. We used a standardised collection sheet prepared for this review detailing the information to be collected thematically (immune processes, gut microbiome, gut-brain axis, probiotics, and fecal microbiota transplant). The authors cross-checked each other’s data sheets, and discrepancies were addressed through discussion. The first author made the final judgement in the rare instance that a consensus could not be reached. Given the variability across study designs and outcomes, a quantitative meta-analysis was not feasible. Therefore, we undertook a structured narrative synthesis in accordance with the Economic and Social Research Council (ESRC) guidance [15].

Quality Assessment

A quality assessment was performed for each article by two authors. The Newcastle-Ottawa Scale [16] was used for cohort and case-control studies, the Assessment of Multiple Systematic Reviews [17] was used for systematic reviews, and the Scale for the Assessment of Narrative Review Articles [18] was employed for narrative reviews. 

Results

**Figure 1:**
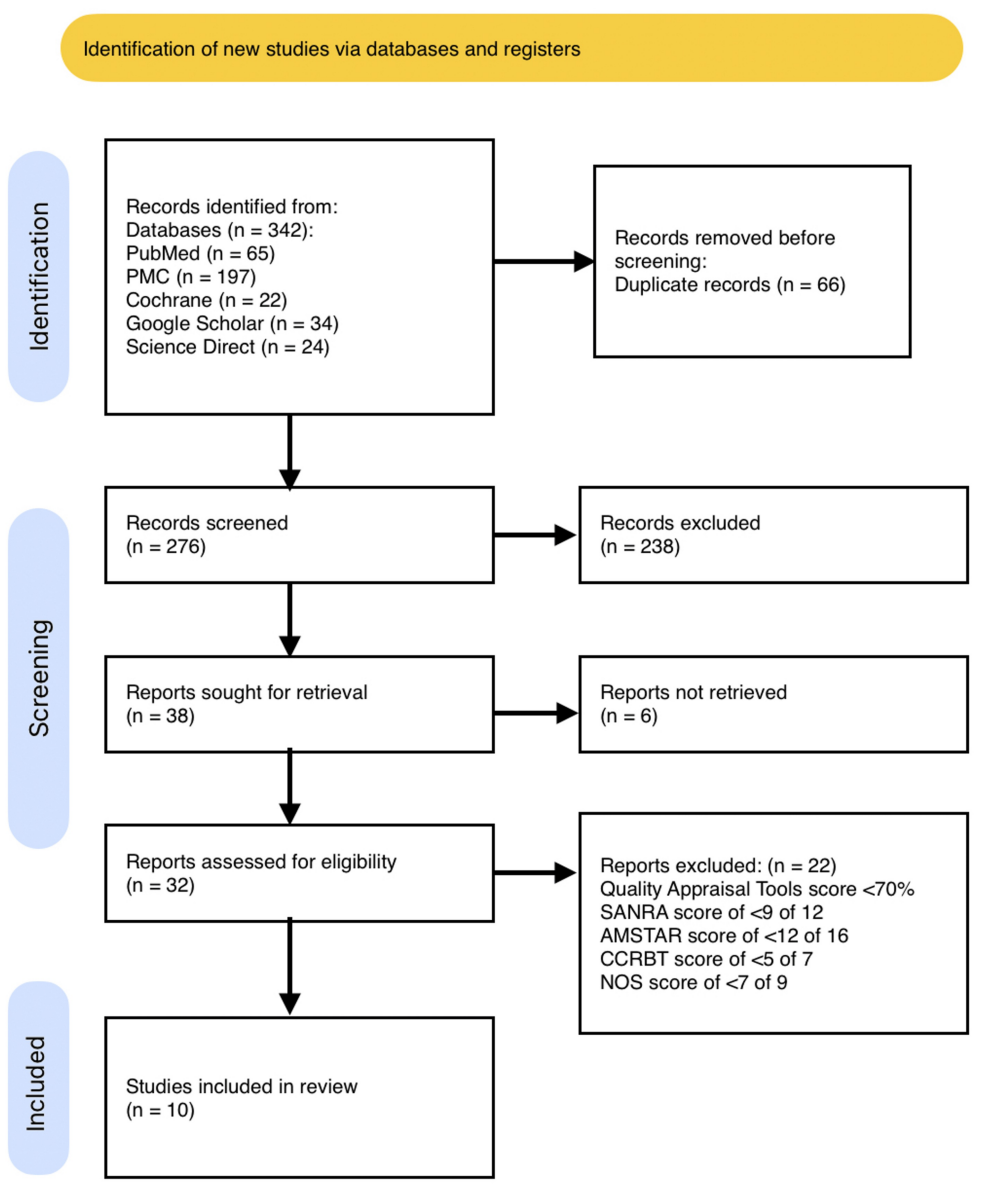
PRISMA flow chart. PRISMA: Preferred Reporting Items for Systematic Reviews and Meta-Analyses; PMC: PubMed Central; NOS: Newcastle-Ottawa Scale; AMSTAR: Assessment of Multiple Systematic Reviews; SANRA: Scale for the Assessment of Narrative Review Articles; CCRBT: Cochrane Collaboration Risk of Bias Tool

The database search revealed 342 potentially relevant titles. Reference browsing was also performed. Sixty-six duplicate records were removed, and 276 were screened based on relevance and the eligibility criteria. Thirty-eight articles remained, but the full text of six could not be retrieved. Thus, thirty-two full-text articles were thoroughly checked, resulting in a narrowing down to 14 papers. The studies were screened based on their titles and abstracts to exclude those that were irrelevant, followed by a retrieval of the full-text articles. Finally, the remaining full-text papers underwent thorough quality evaluation. Ten articles with a score of greater than 70% were included: one case-control study, two cohort studies, one systematic review, and six narrative reviews. The PRISMA chart is provided in Figure 1.

Table 2 contains information regarding the quality appraisal for each type of report, the minimum score for inclusion, and the studies accepted in this review. Table 3 specifies the year of publication of each report, along with the study design and outcomes.

**Table 2:** Quality Assessment NOS: Newcastle-Ottawa Scale; AMSTAR: Assessment of Multiple Systematic Reviews; SANRA: Scale for the Assessment of Narrative Review Articles

Quality assessment tool	Type of study	Total score	Minimum score for inclusion	Number of included studies	Included studies
NOS for cohort	Cohort	8	6	2	Settembre et al., 2022 [2] Labus et al., 2019 [19]
NOS for case control	Case control	9	7	1	Clos-Garcia et al., 2019 [20]
AMSTAR	Systematic review	16	12	1	Valencia et al., 2022 [7]
SANRA	Narrative review	12	9	6	Garofalo et al., 2023 [8] Alfaro-Rodríguez et al., 2024 [10] Shaikh et al., 2023 [12] Singh et al., 2023 [13] Zhao et al., 2024 [21] Chen et al., 2022 [22]

**Table 3:** Summary of articles included in the review FMT: fecal microbiota transplantation; NA: not available; IBS: irritable bowel syndrome; FM: fibromyalgia; NT: neurotransmitter

Reference	Year	Study design	Outcomes
Settembre et al. [2]	2022	Cohort study	Study population: IBS (n=53); FM (n=49); aged between 46-56 years. The prevalence of FM in patients with IBS and the prevalence of IBS in FM patients were evaluated according to the Rome IV criteria. An association between FM and disorders of gut–brain interaction, such as IBS, was confirmed.
Valencia et al. [7]	2022	Systematic review	Study population: IBS (n=439, mean age: 34.6 years); FM (n=33912, mean age: 50.2 years). The common processes in IBS and FM include dysregulated immune and inflammatory responses involving mast cells and cytokines. Changes in NT release also contribute to the symptoms.
Garofalo et al. [8]	2023	Narrative review	Specific bacterial species alterations were identified in FM and IBS. Gut dysbiosis and gut–brain axis alterations, including changed levels of NTs and short-chain fatty acids, may contribute to central sensitization. Probiotics and FMT are potential treatment approaches but require further validation through studies.
Alfaro-Rodriguez et al. [10]	2024	Narrative review	Study population: NA. Disturbances in tryptophan metabolism and low serotonin (5-HT) were noted in FM patients. The kynurenine pathway appears to have an important role and should be assessed in relation to FM symptoms.
Shaikh et al. [12]	2023	Narrative review	Study population: NA. Changes in the gut microbiota and gut–brain axis are associated with IBS. Probiotics and FMT do not show reliable results and need investigation.
Singh et al. [13]	2023	Narrative review	Study population: NA. Alterations in the cross-talk between the brain and the gut microbiome, alongside changes in 5-HT signaling, as well as genetic, immune, and psychosocial factors are implicated in IBS pathophysiology.
Labus et al. [19]	2019	Cohort study	Study population: IBS (n=65, mean age: 33.3 years). IBS patients displayed disturbed gut–brain interactions compared to healthy controls. This may be attributed to the influence of the gut microbiome on serotonergic pathways and may influence pain perception in patients.
Clos-Garcia et al. [20]	2019	Case control	Study population: FM (n=105, mean age: 53 years). Gut bacteria diversity was decreased in FM patients. Changes in levels of NTs, such as glutamate, were also found. Specific bacterial changes were identified. Gut microbiome assessment and serum metabolomics offer insight into FM pathogenesis.
Zhao et al. [21]	2024	Narrative review	Study population: NA. Gut dysbiosis impacts the pathogenesis and severity of IBS. Specific biological changes need to be studied, and regulation of the gut microbiota is a personalized and promising approach for therapy.
Chen et al. [22]	2022	Narrative review	Study population: NA. A bidirectional relationship exists between the gut microbiome and NTs (such as 5-HT and gamma-aminobutyric acid), which influences intestinal movement and immune responses. Exploring this relationship in IBS patients has utility in developing treatment strategies.

Discussion 

Immunological and inflammatory processes in FM and IBS

Abnormal immune functioning with elevated levels of inflammatory cytokines has been implicated in FM and IBS [9,13]. Valencia et al. suggested an imbalance between pro- and anti-inflammatory processes in both conditions [7]. They emphasized that mast cells are activated early in the pathogenesis of both diseases, resulting in the production of pro-inflammatory cytokines, such as interleukins (IL-1 beta, IL-8, IL-6, and IL-17) and tumor necrosis factor alpha (TNF-α), which affect the intestinal mucosa in IBS patients, while their systemic impact may cause widespread body pain in FM patients.

The stress response pathway, known as the hypothalamic-pituitary-adrenal (HPA) axis, acts as the body's central stress management system. When activated by psychological or physical stress, the brain releases corticotropin-releasing hormone (CRH), which triggers a cascade ultimately leading to cortisol production. In both FM and IBS, this stress response becomes dysregulated: High levels of CRH activate innate immune pathways, particularly through mast cells. These immune cells sensitize nociceptors and CRH receptors, which upregulate cytokine production, thereby heightening pain sensitivity and intestinal inflammation. This explains why stress consistently worsens symptoms in both conditions. Additionally, chronic stress perpetuates a vicious cycle: pain and GI symptoms cause stress, which further activates the HPA axis, worsening dysbiosis and inflammation. Liu et al. reported that colonic leptin, a cytokine released from mast cells, is significantly increased in patients with severe symptoms of IBS with diarrhea (IBS-D) predominance [23]. According to Zhao et al., the severity of FM is associated with increased levels of monocyte chemotactic protein 1/chemokine C-C motif ligand 2 [24]. IBS and FM may have developmental processes in distinct locations, but the involved inflammatory cells have a systemic effect that leads to shared symptomatology. This was highlighted by the authors, considering a study in which high levels of plasma TNF-α in IBS patients positively correlated with their complaints of fatigue, a prominent symptom in both IBS and FM [7]. 

Shaikh et al. observed that stress contributes to an increase in pro-inflammatory cytokines in IBS patients, as psychological stress can trigger increased production of inflammatory cytokines through the hypothalamic-pituitary-adrenal (HPA) axis via the release of CRH and, consequently, adrenocorticotropic hormone and cortisol [12]. The authors also cited the TNFSF15 gene as evidence of a genetic component to IBS, since it encodes the protein TL1A, which participates in the activation of the immune cell-mediated inflammatory response in intestinal mucosa. Furthermore, alterations in the gut microbiome may lead to inflammatory and immunologic disruptions that increase gut permeability, thus affecting gastrointestinal homeostasis and, subsequently, the brain-gut pain pathways. This leads to visceral hypersensitivity, which manifests as pain in patients [12]. 

Zhao et al. discussed studies that highlight the influence of interactions between toll-like receptors (TLRs) and specific bacterial cell wall components in IBS [21]. They reported that an elevation in TLR2, TLR4, TLR5, TLR9, lipopolysaccharide (LPS), and anti-flagellin antibodies correlates with IBS symptoms. According to studies referenced by Zhao et al., LPS produced by *Shigella* and nonpathogenic *Escherichia coli* K2 affects the motility of the colon via TLRs. However, the precise mechanism of the impact of LPS on gut motility is not yet ascertained. Additionally, they investigated antimicrobial peptides, such as human defensins, cathelicidins, and lysozymes, which are natural antibiotics produced by various intestinal cells. These peptides modulate interactions between the host and the microbiome and also serve an immune function by killing pathogens. Raised β-defensin 2 levels were recorded in patients with IBS, suggesting immune system activation in the absence of gross inflammation [21]. These findings are consistent with Valencia et al., in highlighting that dysfunctional mast cells are pivotal in IBS, alongside the release of prostaglandins and histamines, which heighten intestinal sensitivity and can lead to abnormal defecation patterns [7].

The immunological and inflammatory processes in IBS and FM share similarities, such as the initiation of the HPA axis by stress, leading to cortisol release, mast cell activation, and cytokine dysregulation. Innate immunity plays a crucial role as TLRs interact with microbiota in IBS, while in FM, monocyte chemotactic proteins and chemokine motif ligands are elevated. The pro-inflammatory cytokine TNF-α is involved in both diseases. While the precise process of instigation to inflammation and the specific locations seem unclear, immune dysregulation notably underlies the symptomatology of both conditions [7,12,13,21].

Gut Dysbiosis and Loss of Gut Barrier Integrity in FM and IBS 

The human gut has commensal microorganisms constituting the enteric microbiome and is widely known to be a key player in maintaining homeostasis and peristalsis, modulating gut inflammation, and synthesizing vitamins and NTs, including acetylcholine (Ach), serotonin, and γ-aminobutyric acid (GABA) [13]. According to Shaikh et al., the gastrointestinal tract is primarily inhabited by the following microbes: Firmicutes (64%), Bacteroidetes (23%), Proteobacteria (8%), and Actinobacteria (3%) [12]. When gut flora are altered due to the depletion or overgrowth of these microbes or due to mutations, it results in dysbiosis. New research has indicated that gut dysbiosis may contribute to the pathogenesis of IBS. Even minor disruptions in the gut microbiome may trigger oxidative stress and damage the integrity of the gut barrier [8,12,13,21]. NT release as a response to stress has been associated with the expression of pathogenic bacteria, such as *Pseudomonas aeruginosa* and *Campylobacter jejuni*. Studies have found abundant *Ruminococcus gnavus* and Lachnospiraceae and lower levels of *Barnesiella intestinihominis* and *Coprococcus catus* in IBS patients [12,25]. An increased Firmicutes to Bacteroidetes ratio has been noted, as well as increased populations of Clostridia and Clostridiales. However, decreased populations of Bacteroidia and Bacteroidales have been observed. Lower levels of *Lactobacillus *and *Bifidobacterium* and higher levels of* E. coli* and *Enterobacter* have been found in IBS patients. No substantial difference has been detected in the levels of fecal *Bacteroides* or *Enterococcus *in IBS patients and healthy controls. A study also recognized the significance of the gut virome in IBS by identifying lower alpha diversity and altered beta diversity in IBS patients, the highest concentrations of which were the Siphoviridae, Myoviridae, and Podoviridae families [25].

Garofalo et al. gathered evidence from numerous studies to elucidate the alteration of microbiota composition in patients with FM and IBS compared to healthy controls [8]. They reported that both FM and IBS patients show reduced populations of Bifidobacteria (which synthesize the NT GABA) and Ruminococcaceae (which produce butyrate). The reduction in Bifidobacteria remains consistent with the findings of Shaikh et al., though they offer opposing documentation regarding Ruminococcaceae [12]. 

*Clostridium scindens* contributes to bile acid synthesis and is increased in both FM and IBS patients. This microbe is believed to convert primary bile acids to secondary bile acids, which may participate in nociception [26]. Bile acids interact bidirectionally with gut microbiota. They are believed to play an important role in gut motility and sensitivity, and they contribute to innate immunity within the intestine with an antimicrobial action, although the precise mechanism is unclear. Reduced bile acid levels create a favorable environment for gram-negative bacteria, which promotes a pro-inflammatory microbial population [27]. Certain Clostridia species have been linked to more severe disease symptoms, such as higher pain perception. Garofalo et al. noted that Eubacteria, which produce butyrate, are reduced in FM and increased in IBS [8]. Lachnospiraceae, which produce butyric acid, have been observed to be reduced in FM patients, while they seem variably increased or decreased in IBS patients. This variation may be attributed to the heterogeneity of the study population and warrants further testing.

Reduced butyrate, however, may cause a drop in the synthesis of short-chain fatty acids (SCFAs), which aid the integrity of the gut and blood-brain barrier. A leaky gut would allow the systemic release of LPS from gram-negative gut bacteria, which may interact with peripheral neurons and the CNS, enhancing pain perception. Lachnospiraceae are especially low in IBS patients reporting anxiety and depression, implying that these bacteria may be involved in the psychological aspects of IBS and FM. Rikinellaceae are bacteria that help digest crude fiber in the gut, and their population has been documented to be increased in FM patients while reduced in IBS patients. Many FM patients have been found to have small intestinal bacterial overgrowth (SIBO), and this supports the idea that the expanded microbes cause the translocation of endotoxins through a compromised intestinal barrier, resulting in inflammation and pain sensitization. Although the precise mechanism remains unclear, SIBO is also hypothesized to play a role in the pathogenesis of IBS [12].

A study by Bednarska et al. showed an increase in the migration of bacteria, specifically *E. coli *and *Salmonella*, to the lamina propria in biopsies of patients with IBS [28]. This migration was enhanced due to the upregulation of vasoactive intestinal peptide and its receptor, vasoactive intestinal peptide adenylate cyclase 1 (VPAC1). Further evidence was gathered when a blockade of receptors (i.e., VPAC1) led to a decrease in the migration of the bacteria and clinical improvement in the symptoms. Additionally, a substantial amount of mast cells and decreased efficiency of the tight junctions in intestinal mucosal cells disturbed the integrity of the gut barrier [7].

As stated by Zhao et al., certain mucus-degrading bacteria can reshape the defensive function of the mucous layer by changing its pH, viscosity, and elasticity, which is of consequence in the colonization of intestinal epithelial cells [21]. *Bacteroides fragilis*, Clostridia, and Enterobacteria emit toxins that dissolve mucosal glycoproteins and enhance mucosal inflammation, facilitating IBS symptoms, such as abdominal pain and diarrhea. They recorded that Lactobacillaceae regulate intestinal permeability and sensitivity and improve cognition through IL-22 signaling, while *Corynebacterium*, *Streptococcus*, and *Enterococcus *spp. increase intestinal permeability and hypersensitivity via the 5-HT pathways. *E. coli*, *Achromobacter liquefaciens*, and *Bacteroides* spp. support mucosal barrier repair through the indole pathway [21].

*Limosilactobacillus reuteri*, *Lactobacillus delbrueckii *subsp. bulgaricus, and *Alistipes onderdonkii *modulate intestinal inflammation via Wnt/beta-catenin and aryl hydrocarbon receptor (AhR) signaling. *Akkermansia muciniphila* and *Clostridium butyricum* enhance gastrointestinal motility through TLR2 signaling. *Bacteroides thetaiotaomicron* releases tryptamine and, consequently, Ach and promotes substance P secretion, which improves peristalsis. Fusobacteria also improve motility and decrease hypersensitivity through the serotonergic pathways. Moreover, *Ruminococcus gnavus* facilitates gastrointestinal transport, and *Lachnospiraceae* stimulate TLR5, thereby supporting the innate immune response [21].

Labus et al. conducted a tripartite analysis to explore associations between gut microbes, particularly Clostridia, the brain, and gastrointestinal sensorimotor function in IBS patients [19]. Their study involved fecal sample DNA extraction, microbial composition assessment, and neuroimaging of 65 IBS patients (46 female) and 21 healthy controls (16 female). They elucidated that organisms belonging to the order Clostridiales, especially Ruminococcaceae and Lachnospiraceae, stimulate the synthesis and release of 5-HT from intestinal ECs and modulate gastrointestinal motility. Their analysis revealed strong associations between gut microbes and somatosensory regions in the brain that impact gastrointestinal sensorimotor function in healthy patients. This association appears to have a protective effect on the gut and is diminished in IBS patients. For instance, *Lachnospiraceae incertae sedi *are linked to rectal pain through their connection to the primary somatosensory cortex (which is involved in pain processing) in healthy controls; this is not prominent in IBS patients. Furthermore, Clostridium XIVa and Coprococcus subnetworks in healthy controls exhibit connectivity with gastrointestinal sensorimotor function, involving the subcortical regions (such as the putamen, caudate, and nucleus accumbens) and the primary and secondary somatosensory regions. This connection is not evident in IBS patients, with only the Clostridium XIVa subnetwork showing positive associations with subcortical connectivity, while the Coprococcus subnetwork displays no significant association. Notably, these microbes are implicated in the modulation of the host’s serotonergic system, and it can be hypothesized that a disruption in the microbiome and its connection to the brain contribute to the symptomatology of IBS [19].

Clos-Garcia et al. reported augmented populations of the microbes *Dorea*, *Roseburia*, *Papillibacter*, and *Subdoligranulum* in FM patients [20]. The number of *Bifidobacteria* is reduced, which reinforces Garofalo et al.’s review findings [8]. *Eubacterium*, *Lachnospiraceae*, *Clostridium*, and *Firmicutes *are also decreased in the microbiomes of FM patients, supporting the findings of Garofalo et al. that these microbes are associated with a healthy microbiome and are engaged in SCFA generation [8]. These SCFAs include butyric acid and butyrate, which aid in maintaining a healthy intestinal barrier and blood-brain barrier; thus, their depletion may disrupt the barriers’ integrity. Furthermore, *Bifidobacterium* and *Lactobacillus* promote the production of GABA [29]. The depletion of these organisms may therefore influence the ratio of glutamate to GABA in favor of glutamate. Elevated glutamate may then enter systemic circulation via the interrupted intestinal barrier and through the blood-brain barrier, which is possibly the mechanism behind the raised glutamate in FM patients’ cerebrospinal fluid (CSF). This provides evidence for an altered gut microbiome as a pathophysiological factor in FM [20].

These studies suggest dysbiosis as a pathophysiological mechanism in IBS and FM. An association with SIBO has been observed in both conditions as well. Depleted *Bifidobacteria* are a consistent finding in FM and IBS, while alterations in Ruminococcaceae and Lachnospiraceae differ across the papers. Certain Clostridiaspecies appear to be associated with increased inflammation and pain perception in both conditions. Dysbiosis affects SCFA and NT synthesis, leading to compromised intestinal and blood-brain barriers, thus allowing for the inappropriate release of cytokines and altered levels of NTs. NT disturbance is an important component in both conditions and thus warrants further discussion.

NTs and the Gut-Brain Axis in FM and IBS 

**Figure 2:**
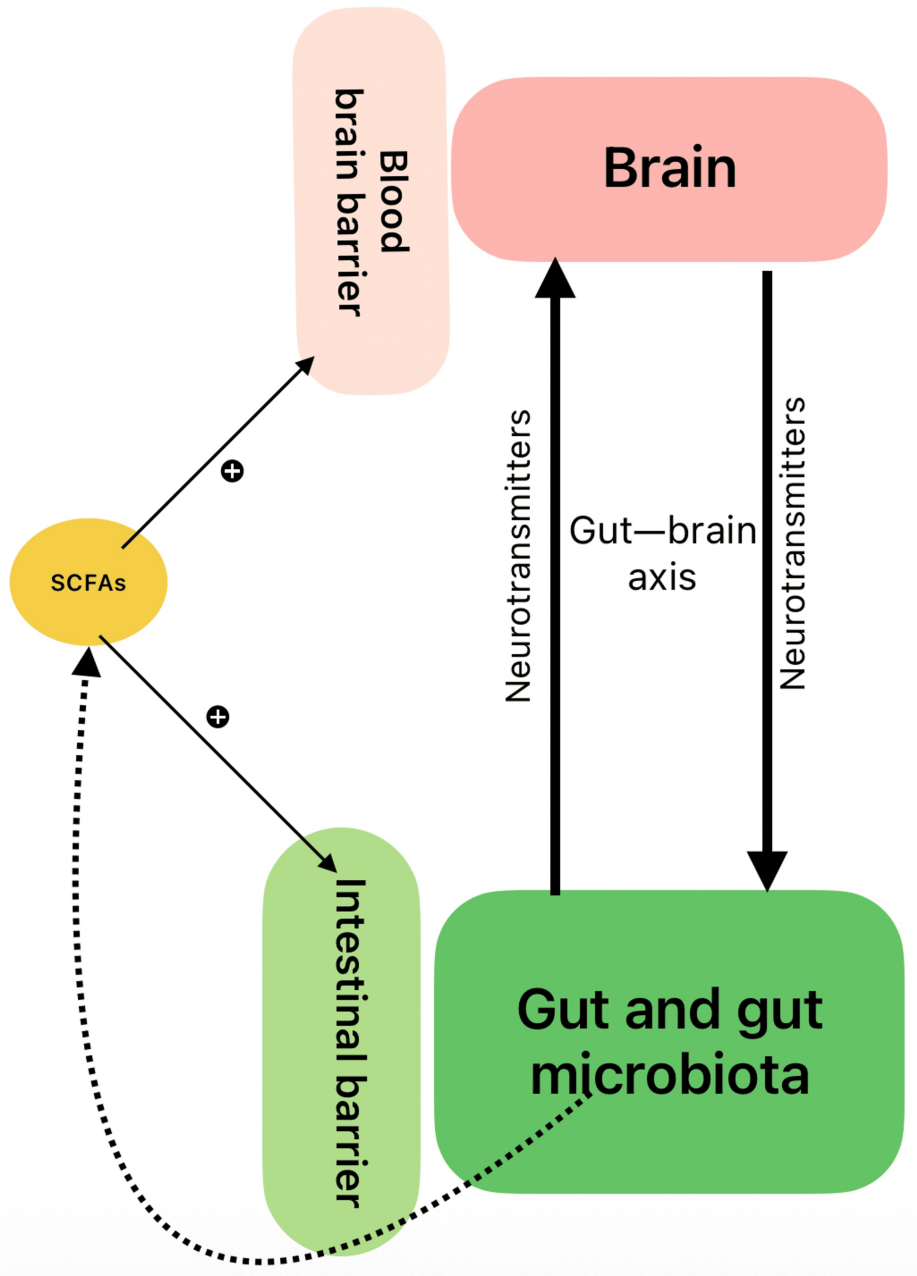
SCFAs, gut-brain axis and gut microbiota. SCFA: short chain fatty acids Image Credit: Roshitha S. Bheemaneni

Gut bacteria and the brain communicate reciprocally through neural, endocrine, and immune pathways that constitute the microbiota-gut-brain axis. Signaling from the gut bacteria occurs via epithelial cell receptor mediators and the direct involvement of lamina propria cells. The brain, on the other hand, exerts itself through the release of signaling molecules in the gut lumen and motility. The vagus nerve is a crucial modulator of this axis and is sensitive to NTs, including histamine, 5-HT, glutamate, GABA, dopamine, Ach, and catecholamines. These NTs are regulated by diet-responsive gut bacteria; specifically, enteric 5-HT is synthesized by ECs in the gut and serotonergic neurons in the CNS. Microbial-associated molecular patterns from the microbiota directly influence the serotonergic system through the modulation of the serotonin transporter and serotonin receptors, in addition to the formation of 5-HT in the gastrointestinal tract; 5-HT mediates bidirectional cross-talk in the gut-brain axis and affects gastrointestinal motility, mucosal permeability, and visceral hypersensitivity [8,13,29,30]. Figure 2 shows a schematic representation of the gut-brain axis. 

Valencia et al. reported findings of abnormal serotonin and dopamine secretion in IBS patients due to dysbiosis. On the other hand, there is a possibility of genetic polymorphisms in serotonin, dopamine, and catecholamine pathway genes in FM [7]. 

Alfaro-Rodriguez et al. elucidated the action of serotonin and kynurenine pathways in FM [10]. They observed that 5-HT levels and 5-HT transporter were significantly lower in FM patients’ CSF. Pain signals are intercepted by 5-HT in the spinal cord by suppressing the release of substance P; therefore, lower levels of serotonin are associated with heightened pain sensitivity. The authors highlighted studies proposing that the downregulation of serotonin receptors, such as 5-HT2A and 5-HT2C, may contribute to chronic hyperalgesia in FM. Recent studies have shown diminished Trp and kynurenine in FM patients, indicating a correlation between these metabolites and the severity of FM symptoms. Cortisol is rapidly released in response to emotional stress, which activates the Trp-kynurenine pathway and reduces Trp availability for 5-HT synthesis. Consequently, decreased 5-HT contributes to pain, a prominent FM symptom. Simultaneously, increased kynurenine levels result in elevated quinolinic acid within the CNS, which may lead to elevated glutamate activity and cause cellular stress and neural inflammation, further contributing to pain, sleep disturbances, and a broader range of symptoms [10].

Singh et al. reported that 90% of total serotonin is produced by gut microbiota, and it binds to 5-HT receptors on the microglial surface, activating inflammatory cytokines that contribute to gut inflammation [13]. They stated that Trp and its metabolites affect inflammation in the CNS and also influence microglia activity in the gut lumen. This is consistent with Alfaro-Rodriguez et al.’s records of FM patients [10]. Furthermore, Singh et al. documented the “inter-kingdom signaling” regulatory pathway in IBS patients, wherein bacteria and gut epithelial cells maintain communication through NTs and peptides [13]. The gut microbiome also relays to the brain through a TLR signaling pathway. Intriguingly, increased 5-HT synthesis is used by the microbiota to modulate the HPA axis. They described findings stating that microbiota, including *Akkermansia muciniphila* Amuc_1100, *A. muciniphila*, *B. thetaiotaomicron*, *Bifidobacterium dentium*, *Clostridium ramosum*, *Corynebacterium* spp., *Enterococcus* spp., *Streptococcus* spp., *E. coli* Nissle 1917, indigenous spore-forming bacteria, *Lactobacillus plantarum* IS-10506, *L. plantarum* PS128, SadA-expressing staphylococci, *Trichinella spiralis*, and *C. jejuni *(pathogens), upregulate the 5-HT pathway. *B. longum*, *Lactobacillus acidophilus*, *Bifidobacterium pseudolongum*, and *Lactobacillus rhamnosus* have been noted to downregulate the pathway. We can infer that dysbiosis would therefore have a huge impact on normal 5-HT signaling in the gut. Evidence of its implication in IBS can be derived from the amplified levels of gut mucosal 5-HT found in such patients, and their symptoms are linked to aspects regulated by the serotonergic system, such as disturbed gut motility resulting in abdominal pain and altered stool frequency. Moreover, antagonism of 5-HT3A alleviates mucosal inflammation, gut permeability, and, consequently, IBS-like symptoms. Singh et al. cited recent studies that have shown a positive correlation between lamina propria mast cells and a spontaneous release of 5-HT in IBS patients. In addition to serotonin, histamine (which is a mast cell product) is known to cause diarrhea when overproduced [13].

Clos-Garcia et al. [20] agreed with Alfaro-Rodriguez et al. [10] and Singh et al. [13] about increased blood-brain barrier permeability as a result of decreased SCFA-producing bacteria, as well as the role of the gut microbiome in the altered levels of 5-HT, dopamine, and GABA in FM patients. Though 5-HT is not known to cross the blood-brain barrier, its precursor Trp can, which emphasizes its potential role in the gut-brain axis [31].

Chen et al. noted that Ach, increased pressure inside the abdominal cavity, or low pH, activates ECs to release 5-HT, triggering intrinsic sensory neurons in the intestinal wall to facilitate secretory reflex and peristalsis in the intestine [22]. Furthermore, exogenous neurons are stimulated by 5-HT to cause pain, discomfort, nausea, and vomiting. Specifically, peristalsis and the secretory reflex stimulated by 5-HT1 and 5-HT4 receptors on submucosal primary afferent neurons, as well as 5-HT3 receptors on primary afferent neurons, may contribute to reflex activity. They highlighted that 5-HT and 5-HT3 receptors in the intestinal mucosa are substantially higher in IBS patients compared to healthy controls, suggesting an impaired 5-HT system in IBS patients. Dopamine is another NT that possesses intestinal receptors (D1, D2, D3, D4, and D5) and is involved in gut motility and permeability functions. Gut microbiota and dopamine metabolism mutually influence each other. As for dopamine dysregulation in the context of IBS, studies have illustrated that IBS with constipation (IBS-C) predominance patients have increased dopamine, while IBS-D patients show no difference. Importantly, levodopa, dopamine agonists, metformin, butyrate, losartan, and imipramine are potentially useful in relieving IBS symptoms via gut-brain axis modulation. IBS patients also display a disturbance in the NT GABA, with decreased GABA, GABA receptors, and glutamate decarboxylase 2, but increased GABA transporter 2. Additionally, elevated histamine levels in the colon and urine of IBS patients are correlated with the severity of symptoms, particularly abdominal pain. Some gut bacteria, such as *E. coli*, can produce histamine, and the receptors H1 and H4 are implicated in visceral hypersensitivity and pain. Therefore, targeting histamine pathways is also a therapeutic implication in IBS. Chen et al. also noted that the enzyme indoleamine 2,3-dioxygenase, which is responsible for the degradation of Trp, is increased in IBS. However, the levels of neuroprotective kynurenic acid (KynA) were diminished, and the ratio of KynA to kynurenine (Kyn), a precursor to the neurotoxic metabolite quinolinic acid, was also reduced [22]. It is worth noting that reduced Trp and kynurenic acid were also documented in FM patients by Alfaro-Rodriguez et al., which highlights a key similarity in IBS and FM [10].

Zhao et al. reported findings expanding on the role of SCFAs in stimulating the release of 5-HT from EC cells, promoting Ach secretion, and thus, augmented peristalsis [21]. In IBS patients, lowered SCFAs have been observed, which contribute to inflammation, impaired Trp metabolism, and Ach receptor activity and potentially result in visceral hypersensitivity. SCFAs possibly exert influence on the gut-brain axis via the vagus nerve, impacting symptomatology, such as pain and anxiety. Elevated 5-HT levels have also been noted in IBS-D patients, alongside reduced serotonin transporter expression. They reinforced the findings from other papers by noting reduced kynurenic acid levels in IBS patients as well. They also acknowledged that the dysbiosis of histamine-producing gut microbes, such as *E.coli* and *Morganella morganii*, may be implicated in the onset and evolution of IBS [21].

Both IBS and FM exhibit NT alterations and disturbed homeostasis of the gut-brain axis. Serotonin has a pivotal role in pain associated with both conditions, with elevated gut serotonin found in IBS patients and depleted 5-HT observed in the CSF of FM patients. Notably, reduced Trp and kynurenic acid levels are diminished in both diseases, and 5-HT is a derivative of these pathways. While histamine activity appears to influence IBS symptoms, glutaminergic neuroinflammation and an imbalance in the GABA to glutamate ratio play a role in FM symptoms. Abnormal SCFA profiles have also been recorded in both conditions. An important consensus is the reciprocal influences of NTs and the microbiome. Therefore, targeting gut microbiome health and NT receptors to maintain an appropriate balance may be a promising pharmacological approach in treating FM and IBS. Studying these components in patients suffering from both conditions may offer key insights into shared pathophysiological mechanisms located in the gut-brain axis, as well as precise NT alterations. 

Utility of Probiotics and FMT in FM and IBS

Probiotics are live microorganisms believed to augment gut health. They have been found to facilitate innate and adaptive immunity, exert anti-inflammatory effects, and influence the enteric system and CNS via opioid and endocannabinoid receptors [32]. It would not be unreasonable to hypothesize that they would impact diseases such as IBS and FM, whose pathophysiology involves immune cells and the gut-brain axis. 

FMT is a technique in which gut microbiota are transferred from a healthy donor to a recipient with dysbiosis, with the objective of restoring a stable microbiome. This has proven to be efficacious in patients with *C. difficile* infections [32]. Interestingly, some case studies have led to the proposal that FMT also has potential clinical applications in other conditions associated with intestinal dysbiosis [33]. We have established that both IBS and FM are linked to a disrupted gut microbiome; therefore, FMT is a therapeutic strategy worth exploring. 

Garofalo et al. documented that certain probiotics (*Lactobacillus*, *Enterococcus*, and *Bifidobacterium bifidum*) alleviate the severity of IBS symptoms [8]. *Faecalibacterium prausnitzii* produces butyrate, which has anti-inflammatory actions and decreases neuronal excitability, thus relieving pain. *C. butyricum* and *Roseburia hominis* seem to decrease visceral hypersensitivity in IBS patients. *Lactobacillus* and *Bifidobacterium* influence the HPA axis and inhibit stress-induced CNS alterations. Probiotics also boost the production of SCFAs, which help relieve abdominal pain via receptor-mediated mechanisms. They aid in balancing pro- and anti-inflammatory cytokines, potentially contributing to pain relief. *L. acidophilus* increases cannabinoid receptor 2 and colonic μ-opioid receptor expression, decreasing pain sensation. In FM patients, *Lacticaseibacillus paracasei *and *B. longum* infantis improve cognitive functions, including decision-making, but do not appear to impact pain, quality of life, or mood disorders. A combination of *L. rhamnosus*, *L. paracasei*, *L. acidophilus*, and *Bifidobacterium bifidus* has been reported to improve cognitive and emotional symptoms in FM, but these findings are inconsistent due to short treatment durations [8].

Singh et al. also acknowledged that while their mechanisms of action have not been precisely understood, prebiotics may have an anti-inflammatory action, and probiotics may support the intestinal barrier and inhibit the colonization of pathogenic bacteria in IBS patients to offer some symptomatic relief. While FMT has a proven benefit in *C. difficile* infection, optimizing it for IBS patients is a challenging task, given that the populations of different microorganisms in a healthy control have not been fully defined [13].

Shaikh et al. noted that while probiotics may offer symptomatic relief from IBS, data across studies are inconsistent to officially recommend them to patients. The long-term efficacy of FMT also requires more research before it can be suggested as a standard treatment [12].

Anecdotal evidence from a case study by Thurm et al. showed that FMT was substantially advantageous for a patient who suffered from FM, IBS, and chronic fatigue syndrome [34]. Their patient had elevated *Candida* and streptococci, which returned to baseline after the FMT treatment. A complete resolution of fatigue and depression was observed, alongside improvement in insomnia and hypersensitivity to touch and noise. Controlled trials are required to confirm these effects and evaluate efficacy. 

These studies suggest that more extensive research is warranted to consider FMT and probiotics as standard treatment strategies for FM and IBS patients.

Limitations

This review has several limitations. First, only studies from the last 10 years were included, and only full free-text articles in the English language were reviewed. This allowed us to access full, recent studies; however, this could have disregarded relevant older studies and may have led to the exclusion of potentially relevant non-English articles. Additionally, animal studies and Mendelian randomization studies were excluded. They could have provided highly detailed explanations of disease mechanisms and genetic predispositions, but were eliminated due to clinical relevance and applicability. Importantly, the subtypes of IBS, including IBS-C, IBS-D, IBS-M (mixed), and IBS-U (unclassified), may have distinct NT imbalances and gut dysbiosis, which were not detailed in this review, as it focused on the broad correlation of the pathophysiology with FM. 

Second, our discussion of the gut-brain axis was restricted to gut dysbiosis and barrier disruption and did not expound on the CNS mechanisms. Since central sensitization and neuroinflammation have been implicated in these conditions, future research should attempt to explore these further as well. Dopamine pathways and 5-HT can potentially be major pharmacological targets because of their significance in disease pathophysiology, and should be investigated in future studies. Further, only adult populations (≥18 years old) were considered, meaning that pediatric-onset FM and IBS were not addressed. Future research should examine whether pathophysiology differs in younger populations. Finally, FM and IBS are known to be more prevalent in women; thus, assessing the reason for this and variations in mechanisms and clinical manifestations based on gender would be essential in subsequent research. We should also address that causality is still not well defined, so continued inquiry into the sequence of immune dysregulation, gut dysbiosis, and NT imbalance is encouraged to distinguish between the instigating factors and consequent responses. 

Despite these limitations, this review illustrated integral similarity between FM and IBS pathophysiology, specifically within the context of gut dysbiosis and the gut-brain axis, and shed light on the need for continued exploration.

## Conclusions

This systematic review demonstrated the shared pathophysiology between FM and IBS, emphasizing the role of gut dysbiosis, immune dysfunction, and NT imbalances in both disorders. Gut microbiome alterations, Trp and 5-HT metabolism disruptions, and glutamate imbalances play crucial roles in disturbing gut permeability and the blood-brain barrier. These appear to allow for the systemic effects of pro-inflammatory cytokines, causing hypersensitivity and pain. Elevated 5-HT in IBS and depleted levels centrally in FM, along with altered levels of SCFAs, support the hypothesis of gut-brain axis dysfunction. Probiotics and FMT may theoretically restore the gut microbiome and alleviate symptoms, but more evidence needs to be gathered on their efficacy and long-term impact. Future research should look into serotonergic pathway-targeted therapies and gender-specific differences in disease presentation. Patients suffering from both IBS and FM should be recruited into study populations to further understand the association and joint presentation of these diseases.

By reviewing common mechanistic pathways, this review offered insights into FM and IBS causation and correlation. As both conditions present with nonspecific symptoms, awareness of these details can motivate physicians to consider a dual diagnosis during the early stages, leading to timely interventions and enhanced management, and significantly reducing the disease burden. The key gaps of the present literature are a lack of sex-stratified mechanistic studies, and robust evidence on cost-effectiveness for microbiome-targeted therapies. Future studies prioritizing multicenter randomized controlled trials and longitudinal cohort studies can examine and build evidence on the causal pathways and validate therapeutic interventions, including probiotics, psychobiotics, and FMT, relevant to FM and IBS.
